# Current Concepts in the Treatment of Giant Cell Tumor of Bone: An Update

**DOI:** 10.3390/curroncol31040157

**Published:** 2024-04-08

**Authors:** Shinji Tsukamoto, Andreas F. Mavrogenis, Tomoya Masunaga, Kanya Honoki, Hiromasa Fujii, Akira Kido, Yasuhito Tanaka, Costantino Errani

**Affiliations:** 1 Department of Orthopaedic Surgery, Nara Medical University, 840, Shijo-cho, Kashihara 634-8521, Nara, Japan; masunaga.t8111@gmail.com (T.M.); kahonoki@naramed-u.ac.jp (K.H.); hiromasa@naramed-u.ac.jp (H.F.); yatanaka@naramed-u.ac.jp (Y.T.); 2 First Department of Orthopaedics, School of Medicine, National and Kapodistrian University of Athens, 41 Ventouri Street, Holargos, 15562 Athens, Greece; afm@otenet.gr; 3 Department of Rehabilitation Medicine, Nara Medical University, 840, Shijo-cho, Kashihara 634-8521, Nara, Japan; akirakid@naramed-u.ac.jp; 4 Department of Orthopaedic Oncology, IRCCS Istituto Ortopedico Rizzoli, Via Pupilli 1, 40136 Bologna, Italy; costantino.errani@ior.it

**Keywords:** giant cell tumor of bone, extremity, pelvis, sacrum, spine, denosumab, curettage, metastasis, malignant transformation, bisphosphonate

## Abstract

Curettage is recommended for the treatment of Campanacci stages 1–2 giant cell tumor of bone (GCTB) in the extremities, pelvis, sacrum, and spine, without preoperative denosumab treatment. In the distal femur, bone chips and plate fixation are utilized to reduce damage to the subchondral bone and prevent pathological fracture, respectively. For local recurrence, re-curettage may be utilized when feasible. En bloc resection is an option for very aggressive Campanacci stage 3 GCTB in the extremities, pelvis, sacrum, and spine, combined with 1–3 doses of preoperative denosumab treatment. Denosumab monotherapy once every 3 months is currently the standard strategy for inoperable patients and those with metastatic GCTB. However, in case of tumor growth, a possible malignant transformation should be considered. Zoledronic acid appears to be as effective as denosumab; nevertheless, it is a more cost-effective option. Therefore, zoledronic acid may be an alternative treatment option, particularly in developing countries. Surgery is the mainstay treatment for malignant GCTB.

## 1. Introduction

**Figure 1:**
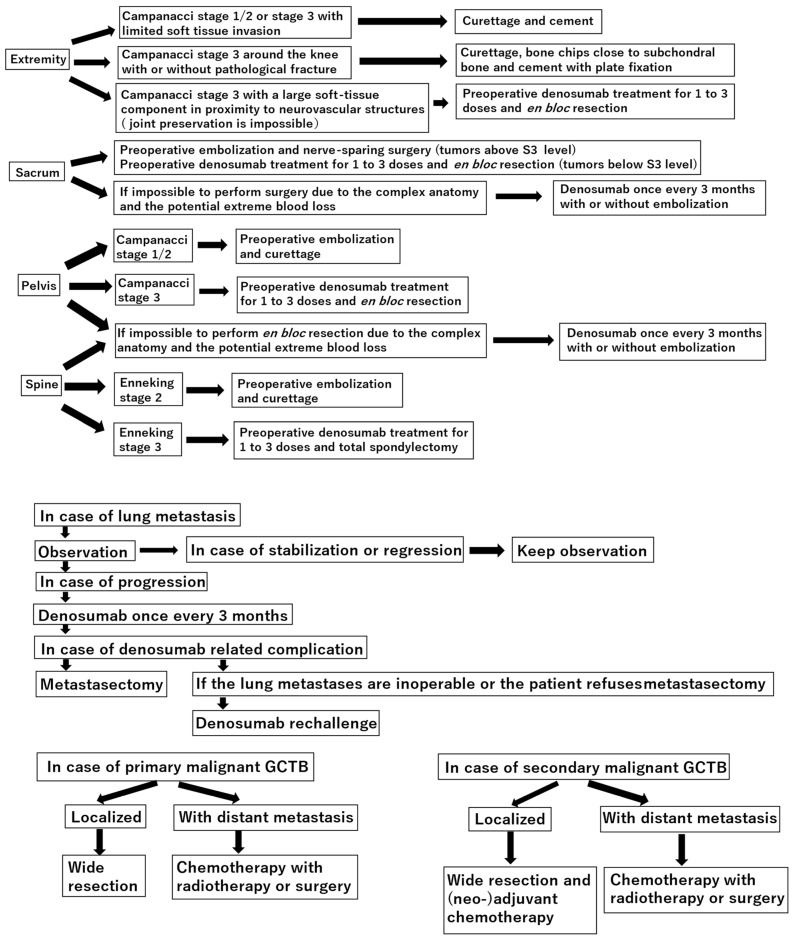
Treatment algorithm for giant cell tumor of bone (GCTB).

Giant cell tumor of bone (GCTB) is a bone tumor of intermediate grade characterized by high local invasive potential, accounting for roughly 5% of primary bone tumor cases [1]. These tumors typically occur at around 30 years of age and are located at the epiphysis [1]. However, tumors may arise at the metaphysis prior to epiphyseal line closure [2]. H3.3 p.Gly34Trp (G34W) immunohistochemistry is a useful diagnostic tool for GCTB [3,4,5]. Lung metastases [6] and malignant transformation occur in 1–9% and 2.4% of patients with GCTB, respectively [7]. Typically, GCTB develops in the distal femur (30%), proximal tibia (28%), distal radius (9%), and distal tibia (6%). However, it is also detected in the pelvis (2%), sacrum (2%), and spine (3%) [8]. Denosumab was approved by the Food and Drug Administration more than a decade ago [9], and it is effective in downstaging to less invasive surgical intervention [10]. At present, this agent is indicated for unresectable GCTB or in case of significant functional impairment following resection [9]. The advent of denosumab altered the treatment strategy for GCTB; thus, it is crucial to update the current therapeutic paradigm for GCTB (Figure 1).

## 2. Methods

We searched for “giant cell tumor of bone” on PubMed, mainly extracting important articles from the literature from January 2021 to March 2024, and described their contents to update the review article published in 2021 [11]. Regarding the literature related to reconstruction after the en bloc resection (EBR) of GCTB of the distal radius, which was not included in the previously published review [11], we cited and described many of the articles published before 2021. Therefore, this review is a narrative review.

## 3. Extremities

For Campanacci stages 1 and 2, curettage is recommended to preserve the joints and to achieve good postoperative function [8,12]. The recommendation indicates aggressive curettage with a sharp curet and a high-speed burr [12,13]. The use of adjuvants (e.g., phenol, ethanol, argon beam coagulator, microwave [14], cautery, and liquid nitrogen) is also recommended [13]. Different types of local adjuvant therapy and subsequent histological evaluation were performed in a porcine humeral and femoral model. The study showed 0.3 mm and 0.8 mm mean depths of necrosis in the phenol and cement groups, respectively. This value was 2.5 mm in the argon beam coagulator, liquid nitrogen spray, and bipolar groups [15].

The prevention of tumor remnants is an important aspect of curettage. Studies revealed no difference in the recurrence risk after curettage performed by orthopedic surgeons trained and not trained in oncology or based on years of experience [16]. The investigators concluded that detailed and careful curettage is more important than oncology training or curettage by a specialist team at a high-volume center [16]. During curettage, it is important to first enlarge the open window to reduce blind spots [12]. In addition, attempts have been made to use open surgery in conjunction with scope to detect tumors hidden in blind spots and prevent residual tumors [17]. Furuta et al. used magnetic resonance imaging during curettage to detect and prevent residual tumors [18]. They reported that the detection rate of residual tumors via intraoperative magnetic resonance imaging was 100% [18].

A recent study investigated liquids used to wash away tumors after curettage, which is typical practice [19]. Moore et al. performed an in vitro evaluation of human GCTB cell lines by exposing them to 0.9% saline, sterile water, 70% ethanol, 3% hydrogen peroxide, 0.05% chlorhexidine gluconate, and 0.3% povidone–iodine solution. The immersion of the human GCTB cell line in 0.05% chlorhexidine gluconate for 2 min resulted in higher cytotoxicity compared with the other liquids (*p* < 0.003). Therefore, the use of 0.05% chlorhexidine gluconate solution for washing after the curettage of GCTB may serve as a chemical adjuvant [19].

Cement, hydroxyapatite, β-tricalcium phosphate, and allograft are the materials of choice for filling bone defects. The advantages of cement include its anti-tumor effect due to heat, ease of recurrence detection on imaging, and early weight bearing [12]. However, disadvantages include the risk of cartilage damage due to cement heat when used in subchondral bone and mechanical failures [20,21]. Jamshidi et al. reviewed 26 patients who underwent allogeneic bone grafting (n = 12) or filling with bone cement (n = 14) after the curettage of GCTB in the proximal femur [22]. Recurrence rates among these two intervention groups were not significantly different (25% and 21%, respectively). Fractures and arthropathic changes were more common in the bone cement group (43%) versus the allograft group (17%) [22]. There are several risk factors for fracture following the curettage of GCTB of the extremities [23]. GCTB in the femur has been associated with a significantly increased risk of postoperative fracture versus GCTB of other sites. Moreover, the presence of a pathological fracture has been linked to a higher risk of postoperative fracture compared with the absence of such fractures. Nonetheless, patients undergoing bone grafting are at a lower risk of postoperative fracture compared with those who do not undergo it [23]. To reduce the risk of fracture, it is recommended to perform bone grafting following curettage in patients who present with GCTB in the femur and a pathological fracture [23]. For such patients who undergo filling with cement and do not receive bone grafting, additional plate fixation is an option [23].

Bone cement exhibits higher stiffness compared with subchondral bone and cartilage, thereby concentrating pressure on these tissues [24,25]. Bone cement is not biodegradable or osseointegrable [26]. A sclerotic rim created through the increased formation of new trabecular bone has been reported. This rim separates the cement from the surrounding bone and subchondral bone layer [26]. Consequently, the shock-absorbing capacity of the subchondral bone layer is decreased [26]. Notably, the use of bone cement to fill a subchondral defect may lead to the occurrence of thermal necrosis of the subchondral bone and articular cartilage [24,27]. Thus, bone cement causes cartilage damage, fractures, and arthrosis [24,27,28]. Filling the subchondral bone with allograft tip bone, followed by the use of cement, is recommended [20].

Takeuchi et al. used calcium phosphate cement for post-curettage bone defects in 26 patients with GCTB and followed them for an average of 87 months [29]. Calcium phosphate cement exhibited excellent, good, and acceptable consolidation into the surrounding bone in 22 patients (85%), 3 patients (12%), and 1 patient (4%), respectively [29]. Local recurrence occurred in three patients (12%). The remodeling of cortical bone defects appeared in 22 patients (85%). The mean Musculoskeletal Tumor Society (MSTS) score was 29 (96%) [29]. Osteoarthritis, chronic synovitis, and fracture all occurred in one patient (4%), respectively, and the conditions were managed with conservative treatment. Calcium phosphate cement provided a biological interface and long-term stability without the need for internal fixation [29]. To address this issue of bone cementation, Tan et al. performed reconstruction with a three-dimensional-printed (3D-printed) strut-type prosthesis for bone defects following the curettage of GCTB in the distal femur of nine patients. During an average follow-up of 31 months, there was no occurrence of local recurrence or postoperative complications. All autografts showed bone fusion at the graft–host junction at an average of 3 months. In addition, excellent osseointegration of the bone/prosthesis interface was recorded at an average of 4 months. The combination of 3D-printed strut prostheses and autograft reconstruction was characterized by good biocompatibility, osseointegration, and subchondral bone protection [30].

**Figure 2:**
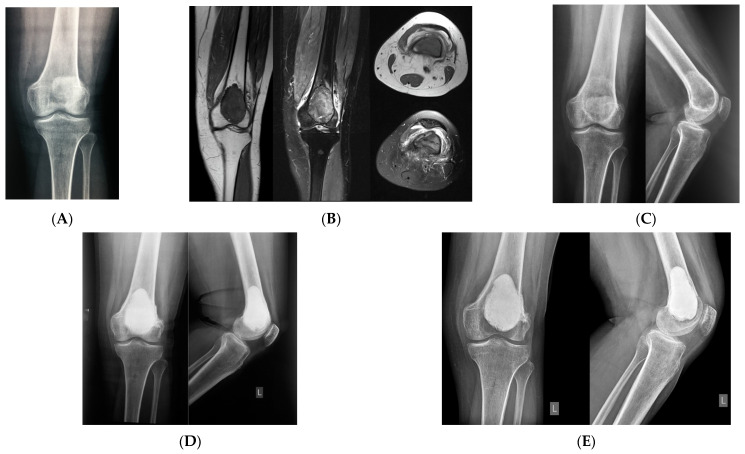

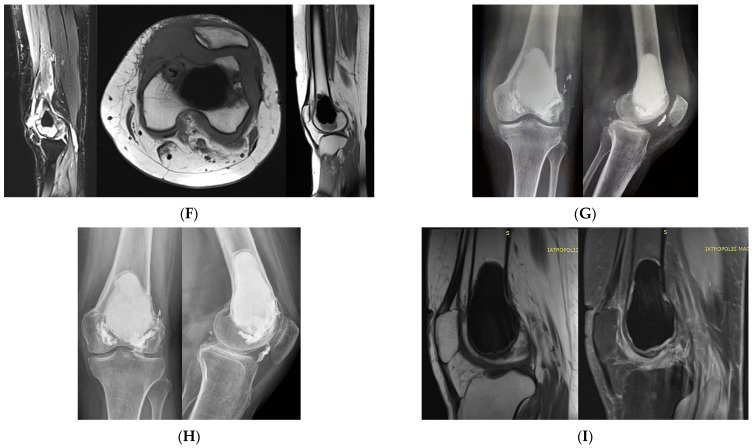
A 49-year-old female patient with giant cell tumor of the distal femur. (**A**) X-ray image captured at presentation. (**B**) Magnetic resonance imaging (MRI) at presentation. (**C**) X-ray image captured after 4 months (7 doses) of preoperative treatment with denosumab. (**D**) X-ray image captured immediately after curettage and cement filling. (**E**,**F**) Local recurrence was observed 2 years after curettage. (**G**) X-ray image captured immediately after radiofrequency ablation and cementoplasty. (**H**,**I**) There was no local recurrence observed at 4 years after radiofrequency ablation and cementoplasty. The patient did not have knee pain.

In GCTB of the extremities, curettage should be performed as much as possible to obtain better postoperative function of the affected limb; this also applies to the treatment of recurrent lesions [31]. Arrigoni et al. performed radiofrequency ablation (RFA) of recurrent lesions after curettage [32]. Of five patients, one had a recurrence 4 months after RFA treatment; the patient underwent EBR and reconstruction using prosthesis, without complications [32]. Minimally invasive RFA is an option for the initial treatment of small recurrent lesions detected during follow-up after curettage, prior to repeat curettage [32] (Figure 2).

The administration of denosumab prior to curettage has been linked to an increased recurrence rate [33,34,35,36,37,38,39]. A systematic review revealed that the recurrence rate in patients treated with preoperative denosumab therapy plus curettage or curettage alone was 20–100% and 0–50%, respectively [34]. The preoperative use of denosumab is linked to bone sclerosis, which complicates curettage and tumor identification. Moreover, it is associated with residual disease, resulting in the reactivation of giant cell tumor cells present in the bone sclerosis lesions following the discontinuation of treatment with denosumab [33,34,40,41]. Denosumab does not cause apoptosis of giant cell tumor cells [42,43]. Studies analyzing the effect of this agent on H3 histone family member 3A (H3F3A)-mutant cells have shown cell persistence after treatment [44,45,46,47,48,49]. Previous studies of GCTB in the extremities demonstrated that preoperative treatment with denosumab was not associated with local recurrence [50,51]. The above studies were retrospective with bias, and causality could not be proved because denosumab was utilized in patients with more aggressive GCTB [34]. The randomized controlled trial (JCOG 1610 study) [52] compared denosumab administration for 2 months before curettage versus curettage alone. However, the study was terminated because of poor patient collection, yielding only descriptive results; consequently, the investigation failed to demonstrate the superiority of preoperative denosumab plus curettage over curettage alone [53]. Based on the above reports, it is not recommended to administer preoperative denosumab before the curettage of GCTB in the extremities of patients in whom joint preservation may be achieved.

The rates of local re-recurrence, joint preservation status, and affected limb function after surgical intervention for locally recurrent disease and preoperative denosumab therapy combined with curettage have been examined [54]. Local re-recurrence was detected in six patients (16%) with challenging joint preservation and six patients (21%) who underwent curettage. The nine patients who underwent EBR did not experience local re-recurrence [54]. Joint preservation was achieved in 24 of the 38 patients (63%), and the median MSTS score was 28 [54]. The follow-up after surgery lasted for a median of 64 months to monitor the development of local recurrence [54]. Therefore, the preoperative administration of denosumab may be considered for patients who require EBR [54]. Preoperative treatment with denosumab plus curettage was evaluated in 25 patients with GCTB who had pathological fractures with only a small amount of subchondral bone and large extraosseous lesions, in whom joint preservation would be difficult [55]. After 57 months of follow-up, local recurrence was noted in 11 patients (44%) [55]. Although preoperative treatment with denosumab increases the risk of recurrence, the re-curettage of recurrent lesions is possible; therefore, the benefit of joint preservation is considered to be greater after re-curettage. Therefore, preoperative treatment with denosumab might be useful in patients in whom joint preservation is challenging.

In GCTB around the knee, age, the distance between the tumor edge and articular surface (<2 mm), and the destruction of the posterior cortical bone have been significantly associated with local recurrence following curettage [56].

The presence of a fluid–fluid level (FFL) suggests s secondary aneurysmal bone cyst (ABC), linked to a higher recurrence rate after curettage [57,58]. Secondary ABC was found in 60 of 256 patients with GCTB in the extremities; the recurrence rate was 53% among patients with secondary ABC versus 26% in those without secondary ABC (*p* < 0.05) [57]. The reason for this difference is that secondary ABCs result in increased blood loss, thereby blurring the operative field and leading to inadequate curettage. ABCs include a blood-filled cavity within a dilated bone segment; the cyst wall is composed of fibrous components, macrophages, giant cells, and bone islands [59,60,61,62]. Approximately 70% and 30% of ABCs are primary and secondary lesions, respectively; these tumors are preceded by primary bone lesions (e.g., fibrous dysplasia, GCTB, osteosarcoma, chondroblastoma, hemangioma) [63]. ABCs arising from GCTBs are the most frequently detected lesions (14–35%) [63,64,65]. FFL occur when a cyst contains material of different densities (liquid) on a compartmentalized structure; the boundary between the two layers is in a horizontal plane at 90° to the direction of gravity [66,67]. They occur when imaging is carried out in a gravity-dependent plane [66]; FFL are present in 16% of patients with GCTB, while secondary ABCs develop in half of patients with FFL [68].

In a retrospective study of 411 patients with primary benign bone tumors who underwent curettage, Zhou et al. demonstrated that blood pressure and the use of tourniquet are associated with local recurrence following the curettage of primary benign bone tumors [69]. In the absence of tourniquet use, the preoperative mean arterial pressure was predictive of local recurrence (*p* < 0.001) [69]. With tourniquet use, the preoperative mean arterial pressure did not show a relationship with local recurrence (*p* > 0.05) [69].

The recurrence rate increases in parallel with the neutrophil-to-lymphocyte ratio (*p* = 0.001) [70] and decreases with an increase in the prognostic nutritional index, calculated as albumin [g/L] + (5 × total lymphocyte count [109/L]) (*p* = 0.003) [71]. A report indicated that the local recurrence rate of GCTB was not associated with the inflammatory markers neutrophil-to-lymphocyte ratio, platelet-lymphocyte ratio, lymphocyte-monocyte ratio, prognostic nutritional index, hemoglobin, alkaline phosphatase, and lactate dehydrogenase [72].

Curettage improves affected limb function compared with EBR (median MSTS score 29.5 vs. 27, respectively, *p* = 0.029) [73]. For GCTB in the distal radius, curettage was associated with significantly lower QuickDASH scores versus EBR and arthrodesis with vascularized fibula graft (13.7 vs. 20.8, respectively, *p* = 0.04) [74]. Good function following the EBR of the fibula and distal ulna is also supportive of EBR, even in cases with Campanacci stage 1 or 2 disease [12]. Zhou et al. compared curettage versus EBR in 28 cases of GCTB in the distal ulna [75]. A significantly higher recurrence rate was observed in the curettage group (n = 7) versus the EBR group (n = 21) (42.9% vs. 4.8%, respectively) [75]. Seven, five, and nine patients underwent the Darrach, original Sauvé–Kapandji, and modified Sauvé–Kapandji procedures with extensor carpi ulnaris tenodesis, respectively [75]. Functions were similar among patients who underwent curettage, Darrach, Sauvé–Kapandji, and modified Sauvé–Kapandji procedures with extensor carpi ulnaris tenodesis [75]. Considering the high recurrence rate following curettage, patients should be knowledgeable regarding the possible benefits/risks of choosing the curettage of GCTB in the distal ulna. Furthermore, reconstruction following the tumor resection of the ulnar head is unnecessary [75].

EBR is indicated for GCTB with large extraosseous lesions at Campanacci stage 3 [12]. GCTB that frequently occurs at the epiphysis requires reconstruction with a megaprosthesis, allograft, or allograft prosthesis composite (APC) after EBR [12]. Various reconstruction techniques are available for the distal radius, such as wrist arthroplasty with proximal fibular head arthroplasty [76,77,78,79], osteoarticular grafting [80,81,82,83], or prosthetic hemiarthroplasty [84,85]. Wrist arthroplasty has the advantage of wrist movement; however, it is linked to the risk of wrist subluxation, pain, and limited pronosupination [76,77,80,84,85]. Wrist fusion with an autograft [77,86] or allograft [87,88] provides long-term wrist stability at the expense of movement [76]. Instability and osteoarthritis have been detected in a proportion of patients following arthroplasty with proximal fibula arthroplasty [77], distal radius allograft [80,81], and custom prosthetic reconstruction [84,85]. Notably, a high revision rate due to allograft fractures was recorded [83,88]. Ulnar translocation with wrist fixation results in a strong and stable wrist [89,90]. In addition, microsurgical techniques (vascular anastomosis) are not required, and the operative time is shorter than that for free vascularized bone grafts [89,90]. The use of an autograft of the iliac crest [91], fibula grafts [76,77,78], or a hemi-cortical strut tibia [86] has been associated with the occurrence of donor site morbidity. Allograft-related concerns (e.g., fusion failure, infection) regarding the use of an allograft for wrist fusion might be mitigated by the reconstruction of the wrist with ulnar translocation [89,90].

Zhou et al. compared curettage with EBR in 51 patients with proximal humeral GCTB [92]. A significantly higher recurrence rate was observed in the curettage group (n = 23) than the EBR group (n = 28) (35% vs. 4%, respectively, *p* = 0.007) [92]. The mean MSTS scores for the groups that underwent curettage, reverse total shoulder arthroplasty with APC, hemiarthroplasty, and arthrodesis were 26, 26, 20, and 23, respectively [92]. EBR and subsequent reverse total shoulder arthroplasty were linked to a lower recurrence rate than curettage and did not result in significant differences in functional outcome scores for proximal humerus GCTB [92]. Therefore, reverse total shoulder arthroplasty with APC might be a reasonable initial therapeutic option for proximal humerus in patients with Campanacci stage 3 GCTB [92]. Most GCTBs are marginally resected with the epiphysis location; consequently, shorter lengths of bone are resected compared with those under the resection of other primary malignant bone tumors (average tumor size: 6.4 cm) [92]. Therefore, reconstruction with reverse total shoulder arthroplasty is particularly suitable in most cases where the deltoid attachment site and axillary nerve can be preserved.

The administration of denosumab prior to EBR of Campanacci stage 3 GCTB may be recommended because it stiffens the tumor and reduces tumor spillover [38,93]. It has been reported that extraosseous lesions shrink with denosumab administration [94]. Preoperative treatment with 1–3 doses of denosumab is recommended, as no difference in recurrence rates between 1–3 doses and >3 doses of denosumab has been reported [95,96]. A systematic review compared local recurrence between patients treated with preoperative denosumab plus EBR and those who underwent EBR alone. The analysis revealed a local recurrence rate of 3.6% (2/56) in the preoperative denosumab and EBR group and 14.2% (40/280) in the EBR alone group (*p* = 0.67) [97]. Preoperative denosumab did not cause a reduction in the proportion of patients with local recurrence among those who underwent EBR [97].

Kanwat et al. retrospectively analyzed patients treated with denosumab (20 patients) or zoledronic acid (ZA) (19 patients) neoadjuvant therapy prior to surgery for GCTB [98]. There were no significant differences in the ossification of lesions, ease of surgery, or recurrence rate [98]. Importantly, ZA was significantly less expensive than denosumab (*p* = 0.001) [98].

## 4. Pelvis and Sacrum

The pelvic region has a complex anatomy, and GCTB is characterized by high local invasiveness. Consequently, a standard surgical technique for pelvic GCTB has not been established thus far. Curettage [99,100,101,102,103,104] or EBR [99,100,102,103,104] are therapeutic options in this setting. Curettage is less invasive, but it is linked to a high recurrence rate ranging from 6% to 43% [99,100,102,103,104]. EBR is associated with low local recurrence rates; however, it is also associated with complications (e.g., infection, hematoma, loss of function, and problems due to pelvic reconstruction) [105,106,107,108,109]. Considering the high local invasive potential of GCTB, tumor recurrence often renders resection unreasonable; therefore, initial surgical treatment is critical. EBR is indicated for lesions with extensive cortical destruction and large soft tissue masses to achieve a safe margin [102,105,110].

Recently, good results of reconstruction with 3D-printed prostheses were reported for reconstruction after EBR. Through a retrospective analysis, Li et al. evaluated seven patients with pelvic GCTB who underwent EBR and reconstruction with a 3D-printed prosthesis [111]. The findings did not reveal local recurrence or distant metastasis (mean follow-up: 35 months) or intraoperative complications [111]. Postoperative radiographs illustrated that the 3D-printed prosthesis matched the shape and size of the bone defect. Moreover, Tomosynthesis-Shimadzu Metal Artifact Reduction Technology resulted in good osseointegration at 3 months (range: 2–4 months) postoperatively [111].

Sacral GCTB is linked to a high recurrence rate after surgery, and sacral nerve root sacrifice leads to markedly lower extremity motor, bowel, and bladder dysfunction [112,113,114]. The preservation of bilateral S3 nerve roots is necessary for normal bowel and bladder functions [115]. To preserve sacral nerve function (particularly S1, S2, and S3), tumors in the cephalic (above the S3 level) and caudal (below the S3 level) portions are commonly managed with curettage (nerve-sparing surgery) and complete resection, respectively [116,117].

Curettage for sacral GCTB is associated with excessive intraoperative blood loss [112]. A reduction in bleeding can be achieved through aortic balloon occlusion [118] and selective arterial embolization [119]. Preoperative treatment with denosumab decreased blood loss during curettage and shortened the operative time [37,119]. Nonetheless, osteosclerosis formed by denosumab may complicate tumor curettage and result in a high recurrence rate after treatment with denosumab [37,119]. Yang et al. found that the mean tumor enhancement rates on contrast-enhanced computed tomography before and at 1, 3, and 6 months after denosumab treatment were 2.14, 1.60, 1.38, and 1.25, respectively; importantly, these rates were no longer significantly decreased at 3 months after treatment [120]. It is not recommended to administer denosumab for >3 months prior to surgery for reducing intraoperative blood loss and facilitating surgery [120]. Liang et al. retrospectively analyzed 66 patients with sacral GCTB who received neoadjuvant therapy with denosumab and underwent nerve-sparing surgery [96]. Patients were classified into an ultra-short course group (≤3 doses, 41 patients) or a conventional group (>3 doses, 25 patients) [96]. The ultra-short course group received a lower dose of neoadjuvant denosumab compared with the conventional group (mean: 2.1 vs. 4.8, respectively, *p* < 0.001) and exhibited a shorter time to surgery (12 vs. 72 days, respectively, *p* < 0.001) [96]. There was less fibrosis and ossification in the former group. Furthermore, operative time (199.9 vs. 187.8 min, respectively, *p* = 0.364) and estimated blood loss (1552.4 mL vs. 1474.0 mL, respectively, *p* = 0.740) were similar. Most patients (95%) received denosumab as adjuvant therapy. Local recurrence was detected in three (9%) and five (21%) patients in each group (*p* = 0.255) (mean follow-up: 29 months). Functional status (motor, urinary, and defecation scores: 25.9 vs. 25.7, respectively, *p* = 0.762) was also similar [96]. An ultra-short course of neoadjuvant therapy with denosumab for sacral GCTB may induce similar radiological and histological responses to those induced via a conventional course [96]. The lower degree of fibrosis and ossification may facilitate nerve-sparing surgery and assist in achieving satisfactory local control and functional status [96].

Treatment with denosumab and embolization can be utilized for patients with inoperable disease or severe dysfunction following operation [121,122,123,124]. In one study, patients with sacral GCTB underwent nerve-sparing surgery or non-surgical treatment (i.e., denosumab combined with embolization, or denosumab only) [125]. The patients were followed-up for a mean of 77 and 51 months, respectively [125]. Of those who underwent operation, 44% experienced recurrence. However, there was no tumor growth observed among patients in the non-surgical treatment group [125]. In the former group, the percentages of continuous disease free (CDF), no evidence of disease, and alive with disease were 56%, 11%, and 33%, respectively. In the latter group, the percentages of CDF and alive with disease were 0% and 100%, respectively [125]. In the nerve-sparing surgery group, postoperative infection, intraoperative bladder laceration, and denosumab-related apical granuloma of the tooth were recorded in 11% of patients. In the non-surgical treatment group, denosumab-related osteonecrosis of the jaw developed in 17% of patients [125]. The mean modified Biagini scores were 0.9 and 0.5, respectively [125]. For sacroiliac GCTB, nerve-sparing surgery is the only intervention that can be employed to achieve CDF. Nonetheless, surgery is associated with a higher risk of complications due to its poorer functional prognosis versus non-surgical treatment [125].

## 5. Spine

The selection of an intervention for GCTB of the spine is based on the Enneking stage [126]. The majority of GCTBs are symptomatic and intracompartmental (active; S2) or symptomatic and extracompartmental (aggressive; S3). Subtotal resection (i.e., curettage) and piecemeal total spondylectomy or total en bloc spondylectomy are suitable for the treatment of S2 and S3 lesions, respectively [126].

A study analyzed patients with GCTB of the spine who underwent operation [127]. Among those with Enneking stage 2 disease, one patient treated with curettage experienced local recurrence. Interestingly, local recurrence did not occur in two patients who underwent total spondylectomy [127]. Of those with Enneking stage 3 disease, eight patients (62%) who received curettage and one patient (9%) who underwent total spondylectomy experienced local recurrence [127].

Zhou et al. compared total en bloc spondylectomy to total spondylectomy with piecemeal resection for GCTB of the spine in Enneking stage 3 cases [128]. The analysis involved 60 patients (mean follow-up: 93 months) [128]. Multivariate analysis demonstrated a significant association between local recurrence and total spondylectomy with piecemeal resection and no adjuvant radiation therapy [128].

Tang et al. retrospectively studied 10 patients with spinal GCTB treated with short-term preoperative denosumab (≤5 doses) and total en bloc spondylectomy [129]. After preoperative adjuvant treatment with denosumab, new ossification was observed in nine patients, and cortical integrity occurred in five patients [129]. A >10% reduction in soft tissue mass was recorded in four patients [129]. In this study, the mean operative time and mean estimated blood loss were 575 min and 2790 mL, respectively [129]. No obvious intraoperative adhesions to the dura mater or major blood vessels were observed. Tumor collapse or breakage did not occur during surgery. The patients did not experience deterioration of neurological function following the operation. In addition, tumor recurrence was not observed during a period of 24 months [129]. Short-term preoperative treatment with denosumab might produce radiological and histological responses that may facilitate total en bloc spondylectomy by stiffening the tumor and reducing adhesions to segmental vessels, major vessels, and nerve roots. This therapy was beneficial in achieving optimal oncologic and functional outcomes [129].

For Enneking stage 2 tumors, curettage is recommended. For stage 3 lesions, denosumab should be administered preoperatively to shrink and solidify the extraosseous lesion, so as to prevent spillover before total spondylectomy is performed [93,129].

## 6. Lung Metastasis

Pulmonary metastasis occurs in 1–9% of cases [6]. Patients with distal radius [130,131], Campanacci stage 3 lesion [132], the presence of pathological fractures [133], and repeated local recurrences [132,134,135,136,137,138] are at an increased risk of developing pulmonary metastases. Treatment with denosumab does not prevent the development of lung metastasis [134]. A systematic review investigated 242 patients with lung metastases from GCTB. The researchers reported spontaneous regression in 4.5% of patients [139]. Of those who developed pulmonary metastases, 45% (10/22) were initially managed through observation; in these patients, the disease remained stable [140]. Hence, observation is recommended for initial management [135,136,140,141]. However, nodules measuring >5 mm in size are prone to enlargement and should be closely monitored [140]. For those that enlarge, denosumab is administered every 3 months [142]. Denosumab is able to halt the progression of lung metastases [94,143]. Metastasectomy is performed in the case of complications related to treatment with denosumab [140]. In patients with inoperable disease or refusal of surgery, denosumab therapy should be re-initiated [140,144,145,146] or stereotactic body radiation therapy should be performed.

A systematic review analyzed patients with GCTB and operable lung metastases who underwent metastasectomy versus those who did not. Of the 138 patients analyzed, 62% underwent metastasectomy, whereas the remaining 38% did not undergo it [6]. Similar mortality rates were noted between the two groups [6], indicating that metastasectomy may not reduce mortality in this setting. Based on this evidence, treating physicians should balance the risks and benefits of metastasectomy for patients with GCTB and lung metastases [6].

## 7. Multicentric GCTB

Multicentric GCTB mainly affects young people (mean age: 22 years; range: 10–62 years) and generally presents as an asynchronism tumor [147]. In a study, the mean interval between the primary and subsequent lesions was 7 years [147], and synchronous lesions were detected in a third of the patients [147]. Lesions most frequently developed in the knee, with the majority located on the ipsilateral extremity [148]. Patients were mainly treated with curettage. Local recurrence and distant metastasis were observed in patients. Multicentric GCTB is uncommon and characterized by an unpredictable course [147]. Continuous monitoring for the occurrence of additional GCTB, especially in the ipsilateral extremity, is essential [148].

## 8. Denosumab Monotherapy

Denosumab monotherapy may be an alternative to surgery in the case of intolerable high invasiveness associated with EBR or unacceptable loss of function that occurs following surgery with adequate margins [9,149]. Denosumab monotherapy was administered to 54 patients with metastatic/unresectable GCTB; 40% (4/10) of those followed-up for a median of 15 months after the discontinuation of denosumab experienced tumor regrowth (median: 8 months) [143]. Nonetheless, denosumab treatment could be repeated; the treatment was effective [140,144,145,146], and all symptoms were relieved via bone formation and possible tumor shrinkage [150]. In a Phase 2 study, in which 532 patients with GCTB were treated with denosumab (120 mg once per month (median follow-up: 58.1 months), the side effects of denosumab monotherapy were hypophosphatemia (5%), osteonecrosis of the jaw (3%), anemia (2%), atypical femur fracture (1%), and hypercalcemia (1%) [149]. In a retrospective study, Jiang et al. did not find statistically significant differences in progression-free survival between patients treated every month (n = 26) and every 3 months (n = 14) [142]. Longer dosing intervals of denosumab for GCTB and standard dosing resulted in similar tumor control [142]. Therefore, extending the dosing period (120 mg every 3 months) is recommended to reduce the incidence of complications.

Denosumab is contraindicated for pregnant patients. Moreover, the long-term impact of denosumab on the childbearing potential of patients remains to be determined [151]. Importantly, GCTB typically develops in women of childbearing age; hence, there is a need for further investigation. Chandler et al. [152] published a case of GCTB in a patient receiving secukinumab for the treatment of psoriatic arthritis, which demonstrated significant findings for intralesional calcifications. Histological analysis identified ossification, new bone formation, and remodeling [152]. In addition, there was a paucity of osteoclast type giant cells [152]. Secukinumab is linked to markedly milder adverse effects (e.g., nasopharyngitis, headache, nausea, diarrhea, and pyrexia) compared with denosumab and is not contraindicated for pregnant patients [153,154]. Thus, secukinumab is a potential alternative to denosumab.

Yue et al. compared denosumab with ZA in patients with unresectable GCTB [155]. Patients were treated with subcutaneous denosumab (denosumab group: 120 mg every 4 weeks; n = 80) or intravenous ZA (ZA group: 4 mg every 4 weeks; n = 80) [155]. Denosumab and ZA resulted in similar tumor responses (*p* = 0.118) and clinical benefits (*p* = 0.574) [155]. A smaller number of patients in the denosumab group (12.5%) versus the ZA group (15.0%) experienced disease progression [155]. Denosumab was linked to fatigue (*p* = 0.001) and back pain (*p* < 0.0001), while ZA was associated with hypocalcemia (*p* < 0.0001), flu-like symptoms (*p* = 0.059), and hypotension (*p* = 0.059) [155]. ZA was significantly more cost-effective than denosumab (*p* < 0.0001) [155]. The cost of managing side effects that occurred during treatment was similar in the ZA and denosumab groups (*p* = 0.425) [155]. At 4 years, the cumulative recurrence-free survival rate was higher in the denosumab group than in the ZA group (*p* = 0.035) [155]. For the treatment of surgically unsalvageable GCTB, denosumab is a safe but expensive option compared with ZA [155].

GCTB has good radiosensitivity; nevertheless, there is a 30% risk of malignant transformation [156,157]. Therefore, van der Heijden et al. suggested limiting the use of radiation therapy to patients with residual or recurrent GCTB (e.g., spinal or sacral sites) for which surgery is unacceptable, denosumab is contraindicated or unavailable, and the lesion is unresectable and uncontrolled even with embolization [158].

The addition of sunitinib to denosumab treatment led to the complete disappearance of multinucleated giant cells and mononuclear stromal cells in one patient [159]. In vitro studies emphasized that denosumab plus lenvatinib is a potentially effective treatment option for GCTB [160]. Through a univariate analysis of 46 patients with GCTB, Metovic et al. observed an increased risk of relapse in those with positivity for programmed cell death-ligand 1 (PD-L1) [161]. A multivariate analysis of 128 patients with GCTB in the spine revealed a significantly increased risk of tumor growth in those exhibiting positivity for programmed cell death 1 (PD1) [162]. Thus, immune checkpoint inhibitors may also be effective against GCTB. Additional studies are expected in the future.

## 9. Malignant GCTB

Malignant GCTB, whether primary or secondary, accounts for around 4% of GCTBs (1.6% and 2.4%, respectively) [7,163]. The simultaneous detection of sarcoma and GCTB indicates primary malignant GCTB. Secondary malignant GCTB is recognized based on the detection of malignancy at the tumor site after surgery or radiotherapy [163]. Of note, the detection of secondary malignant GCTB using imaging is difficult [163]. The mortality rate associated with primary and secondary malignant GCTB is 16% and 63%, respectively [164,165]. In a study, secondary malignant GCTB was detected in half of the patients following curettage [163]. In the remaining patients, secondary malignant GCTB was confirmed through biopsy [163]. Imaging revealed secondary malignant GCTB Campanacci stage 3 lesions in almost all patients (19/20) [163]. Secondary malignant GCTB is also characterized by a long time to relapse [163]. Local recurrence is an independent poor prognostic factor for malignant transformation in patients with GCTB untreated with radiotherapy [166]. The median time from last surgery to local recurrence/malignant transformation was longer than that to local recurrence in benign GCTB (15.2 years vs. 1.3 months, respectively) [166]. Late local recurrence is linked to an increased risk of malignant transformation [166]. Metastasis to the lungs has also been associated with malignant transformation [163].

The reported cumulative incidence of secondary malignant GCTB in patients without prior radiotherapy or administration of denosumab was 0.6% [7]. Eighteen cases of malignant transformation during and after the administration of denosumab have been reported [9,10,35,44,55,167,168,169,170,171,172]. In a study of 526 patients with GCTB who received denosumab, four patients (0.8%) developed malignant transformation [149]. The interval between the diagnosis and malignant transformation of GCTB was 17 months to 11 years [173]. Similar incidence rates of malignant transformation have been reported between patients who received denosumab and those who did not receive it [149]. The long-term monitoring of patients with GCTB receiving denosumab therapy is necessary to determine the safety profile of this agent.

In five of six patients with secondary malignant GCTB, malignant transformation occurred within 1 year of denosumab therapy [174]. Similar to other high-grade sarcomas, the clinical course of secondary malignant GCTB tends to exhibit rapid progression and aggressiveness. Therefore, close clinical and imaging observation is important during the first year following treatment with denosumab for GCTB [174]. After treatment with denosumab, tumor enlargement ceases in 99% of cases [9]. Hence, the occurrence of tumor enlargement during denosumab therapy is suggestive of malignant transformation; in such cases, a biopsy should be considered.

For patients with localized malignant GCTB, wide resection is the recommended therapeutic option. Importantly, the usefulness of adjuvant chemotherapy in this setting is uncertain [163,175]. The mortality rate among patients with primary malignant GCTB without distant metastases who underwent surgery plus adjuvant chemotherapy and surgery alone was 40% and 33%, respectively [176]. Among those with secondary malignant GCTB without distant metastases, this rate was 30.6% and 62.2%, respectively [176]. At present, there is no evidence regarding the effectiveness of adjuvant chemotherapy for treating primary malignant GCTB without distant metastasis. However, it has been shown that this treatment improves the survival of patients with secondary malignant GCTB without distant metastasis [176].

Palliative chemotherapy, radiation therapy, and surgery are recommended for the treatment of malignant GCTB with distant metastases [163,175,177].

## 10. Genomic Profiling

H3F3A encodes the H3.3 protein. GCTB has the following genetic characteristics: mutations in H3F3A are highly specific to GCTB, with the G34W mutation being the most common [1,178]. The H3.3 G34W mutation is specific for GCTB, and almost all histological mimics lack it [3,4,5]. The loss of H3.3K36me3 in mutant H3.3 alters the deposition of repressive H3K27me3 that marks intergenic to genic regions beyond the H3.3 region. This modification promotes other chromatin marks and aberrant transcription, altering the cell fate of mesenchymal progenitor cells and preventing differentiation [179]. Previous studies have shown that H3F3A mutations are also detected in malignant GCTB [4,180]. However, some malignant GCTBs are found to be H3F3A-negative, even if the paired GCTB components are found to be H3F3A mutation positive [166,172,180]. Other reports suggested that TP53 mutations, KRAS/HRAS mutations, TERT mutations, KDM4B/KDM6A loss, and H3K27me3 loss were associated with malignant progression of GCTB [181,182,183]. Furthermore, genetic alterations in the MAPK signaling pathway and potential target fusion genes (BRAF, ALK) have been reported in malignant GCTB lacking H3F3A mutations [184]. It has been suggested that targeted therapy may be effective in such cases [184].

## 11. Research Implication

Denosumab treatment prior to curettage should be considered in patients with GCTB of the extremity who are at Campanacci stage 3 and for whom joint preservation is difficult, although the risk of local recurrence may be increased. Even if recurrence occurs, re-curettage may allow joint preservation. For GCTB of the proximal humerus, en bloc resection and reverse shoulder arthroplasty may be a good indication because function remains the same compared to curettage. Denosumab therapy (every 3 months) is a good option for inoperable GCTB of the pelvis, spine, and sacrum, as well as for growing lung metastases, and it is effective when repeated after discontinuation due to complications.

## 12. Conclusions

Curettage is recommended for the treatment of Campanacci stages 1–2 GCTB in the extremities, pelvis, sacrum, and spine, without preoperative denosumab treatment. In the distal femur, bone chips and plate fixation can be used to reduce damage to the subchondral bone and prevent pathological fracture, respectively. For local recurrence, re-curettage may be used when feasible. EBR is an option for very aggressive Campanacci stage 3 GCTB in the extremities, pelvis, sacrum, and spine, combined with 1–3 doses of preoperative denosumab treatment. Denosumab monotherapy once every 3 months is currently the standard strategy for inoperable patients and those with metastatic GCTB. However, in the case of tumor growth, a possible malignant transformation should be considered. ZA appears to be as effective as denosumab; nevertheless, it is a more cost-effective option. Therefore, ZA may be an alternative treatment option, particularly in developing countries. Surgery remains the mainstay treatment for malignant GCTB.
